# Client satisfaction with family planning services in the area of high unmet need: evidence from Tanzania Service Provision Assessment Survey, 2014-2015

**DOI:** 10.1186/s12978-018-0566-8

**Published:** 2018-07-16

**Authors:** Deogratius Bintabara, Julius Ntwenya, Isaac I. Maro, Stephen Kibusi, Daniel W. Gunda, Bonaventura C. T. Mpondo

**Affiliations:** 1 Department of Public Health, College of Health Sciences, The University of Dodoma, P.O Box 395, Dodoma, Tanzania; 2 Department of Global Health Entrepreneurship, Division of Public Health, Graduate School of Tokyo Medical and Dental University, 1-5-45 Yushima, Bunkyo-ku, Tokyo, 113-8519 Japan; 3 Department of Internal Medicine, Catholic University of Health and Allied Sciences, P.O. Box 1464, Mwanza, Tanzania; 4 Department of Internal Medicine, College of Health Sciences, The University of Dodoma, P.O Box 395, Dodoma, Tanzania

**Keywords:** Client satisfaction, Family planning, Service readiness, Tanzania

## Abstract

**Background:**

Client satisfaction has been found to be an important factor for the uptake and continuation of family planning services. This study aimed to examine the current status of and factors associated with client’s satisfaction with family planning services in Tanzania, which has a high unmet need for family planning.

**Methods:**

The study used data from the Tanzania Service Provision Assessment survey of 2014–2015. A facility was classified as having high service readiness for FP if it scored at least 67.7% on a composite score based on three domains (staff training and guidelines, basic diagnostic equipment, and basic medicines), following criteria developed by the World Health Organization. The exit interview questionnaire was used to collect information from women about their level of satisfaction, whether “very satisfied,” “more or less satisfied,” or not satisfied with the services received. The response was dichotomized into “Yes” if the woman reported being very satisfied with services received otherwise coded as “No”. Unadjusted and adjusted logistic regression models were used to assess the association between the client satisfaction and covariate variables; service readiness, facility type, managing authority, location, management meetings, supervision, provider’s sex, and working experience, clients’ age and education. All analyses were weighted to correct for non-response, disproportionate and complex sampling by using the “SVY” command in Stata 14.

**Results:**

Out of the 1188 facilities included in the survey, 427 (35.9%) provided family planning services. A total of 1746 women participated in observations and exit interviews. Few (22%) facilities had a high readiness to provide family planning services. While most facilities had the recommended equipment available, only 42% stocked contraceptives (e.g. oral pills, injectable contraceptives and/or condoms). Further, trained staff and clinical guidelines were present in only 30% of services. Nevertheless, the majority (91%) of clients reported that they were satisfied with services. In the multivariate analysis, a high service readiness score [AOR = 2.5, 95% CI; 1.1–6.0], receiving services from private facilities [AOR = 2.3, 95% CI; 1.1–5.0], and being in the age group 20 to 29 years [AOR = 0.3, 95% CI; 0.1–0.7] were all significantly associated with clients’ satisfaction with family planning services.

**Conclusion:**

There is a high level of client satisfaction with family planning services in Tanzania. Maintaining and exceeding this level will require improvements in the provision of staff training and the availability of contraceptives in existing services.

## Plain English summary

Client satisfaction is a crucial indicator that measures the extent to which a client is gratified with the services received from healthcare providers. Several studies pointed out that client satisfaction is among the factors which influence the use of family planning services. In this study, we examine the current status of and factors associated with client satisfaction with family planning services in Tanzania, which has a high unmet need for family planning. The exit interviews were performed to family planning clients for providing their opinions on the services they had received. A total of 1746 study participants were involved in this study. Since this survey employed several stages to obtain study participants, the estimates were adjusted during analysis to minimize errors due to complex sampling.

The results presented here indicated the high proportion of clients who had satisfied with the services received. The possible factors identified for client satisfaction with family planning services were; readiness of the facility to provide the service, service provided by privately-owned facilities and client’s age. In conclusion, there is high client satisfaction with family planning services in Tanzania. However, there is a need for improving services provided by publicly-owned facilities in order to increase and sustain client satisfaction.

## Background

Rapid population growth resulting from high fertility rate and reduced mortality has become a new challenge of concern in sub-Saharan Africa (SSA) [1]. Projections show that the population in SSA will have increased by 128 and 290% by the year 2050 and 2100 respectively [1, 2]. Family planning (FP) is among the effective strategies that not only slow the population growth but also reduce maternal and child mortality due to outcomes of unintended pregnancies [2–4]. This led many developing countries in the 1960s to establish several programmes that promotes FP use [2, 5]. Between 1960 to the year 1996, the number of developing countries providing FP services had increased from 2 to 115 [6].

Despite the global emphasize on FP use so as to reduce the burden of overpopulation, Tanzania is still placed among the SSA countries with high fertility rate (5.2 births per women) [7], that is above the African average of 4.7 births per woman reported in 2015 [8]. In response, the Ministry of Health, Community Development, Gender, Elderly and Children (MoHCDGEC) of Tanzania developed the National Family Planning Costed Implementation Program (NFPCIP) which was officially launched in March 2010 [9]. The main goal of the program was to increase the contraceptive prevalence rate among women of reproductive age from 28% in 2010 to 60% by the year 2015. Despite this effort, the target has not been met in Tanzania; as the current uptake of any modern contraceptive (that includes; female sterilization, intra-uterine device, pills, injectable, implants and male condoms) is still low (32%) [7]. Among the reasons for this low uptake of contraceptive is client satisfaction with FP services provided within the health facilities [10–12]. Therefore assessing client satisfaction will help to understand the quality of FP services offered as the crucial aspect for improving utilization of FP services.

Generally, quality of healthcare can be assessed by using several measures but all of them falls under four broad attributes: structure, process, outcome and client experience [13, 14]. Structure; that assesses characteristics of care setting, process; that assesses the service provided to patients, outcome; that evaluate patient health as the results of care received, and client experience; that provides feedback on patient experiences of care. Since the satisfaction explains client experience and opinion regarding services received, this measure help to inform how facility and provider meeting clients’ expectations. Therefore, it is considered as an important measure for assessing the level of services provided by facility or provider [15–17].

Despite the fact that several studies examining client satisfaction with FP services in SSA [10, 18–20] yet, only limited studies explored the relationships between structural (facility organizational characteristics and setting) factors and clients satisfaction. But some factors have been explained to influence the client satisfaction which such as waiting time, kind of provider, privacy, staff motivation, poor quality of care, availability of medicines and equipment, and some demographic characteristics like age and education of client [10, 19, 21]. However, some factors such as the type of managing authority and readiness of facility to provide services were left unstudied, that we think they might have some contribution to client’s satisfaction. Therefore, this study aimed to examine the current status of and factors associated with client satisfaction with FP services in Tanzania, which has a high unmet need for FP services. The obtained findings may provide a broad spectrum of policymakers, and public health specialists to design appropriate interventions for strengthening the quality of FP services.

## Methods

### Data source

This study analyzed data from the 2014–15 Tanzania Service Provision Assessment (TSPA) Survey dataset. This survey was undertaken by Tanzania’s National Bureau of Statistics (NBS) in collaboration with the Office of the Chief Government Statistician (OCGS), Zanzibar, the Ministry of Health, Community Development, Gender, Elderly, and Children (MoHCDGEC), Tanzania Mainland, and the Ministry of Health (MOH), Zanzibar. The technical assistance for the survey was provided by ICF International through the United States Agency for International Development (USAID)-funded Demographic and health survey (DHS) program.

### Sample size and sampling procedure

The 2014–15 TSPA was a sample survey of all the formal sector health facilities in Tanzania. The MoHCDGEC in Tanzania mainland and the Ministry of Health (MOH) in Zanzibar provided the list that included all the hospitals, health centers, dispensaries, and clinics. The list consisted of 7102 verified (active) health facilities and used as a sampling frame. From the list, a total of 1200 facilities were randomly selected for inclusion in the survey. The sample was designed to provide nationally representative results according to the facility type, managing authority, and regions for both Tanzania Mainland and Zanzibar. Though of 1200 health facilities sampled, seven refused to participate, four had closed down, and one could not be reached. Finally, a total of 1188 facilities were successfully surveyed. But, the analysis for this study was based on 427 health facilities that reported to provide FP services. These included 106 hospitals, 34 health centers, and 287 dispensaries/clinics. Thereafter, a total of 1746 women clients who received FP services were systematically selected in FP exit interviews based on the number of clients present at service site on the day of the visit. In case many clients were present and eligible for the interviews, a maximum of five clients for each provider and 15 clients for each selected facility were observed. Then all observed clients were included in an exit interview soon after receiving FP services from providers.

### Data collection

The 2014–2015 TSPA survey data were collected between October 20, 2014, and February 21, 2015, and revisit of some facilities that were not covered previous were conducted between March 2 to 13, 2015. The exit interviews were performed by health workers (nurses) selected from different institutions trained and qualified to be interviewers. The 2014–2015 TSPA used four main types of data collection tools: facility inventory questionnaire, health provider interview questionnaire, and observation protocols for antenatal care (ANC), FP, and services for sick children, exit interview questionnaires for ANC and FP clients and for caretakers of sick children whose consultations were observed. The information collected for each questionnaire is stored in a different file.

### Data processing and management

The current study used data from Facility inventory, Health Provider, and Observation/client files. The data in these files were edited and cleaned and later were merged together into the new file (dataset) by using the facility and provider identification as a unique identifier of these three files. During merging process, the Observersion/client file was used as the master file, while the others as using files. The merge command in Stata was performed at M:1 (many-to-one); therefore, the unit of analysis of the merged file remained at the client level.

### Definition of variables

#### Outcome variable: client satisfaction with FP services

##### Client satisfaction

This was the primary outcome variable in which during the exit interviews client was asked to provide their opinion about the FP services they either received or provided at that facility on the day of the interview. The responses were “very satisfied,” “more or less satisfied,” and “not satisfied” with the services received. Later, the responses were dichotomized into “Yes,” if the client reported “very satisfied” with the FP services received or provided and “No,” if client reported either “less or more satisfied” or “not satisfied” with FP services received or provided.

### Key independent variable (proximate factor)

#### FP service readiness

##### FP services readiness

This variable was measured based on the score of FP service readiness index. The score was constructed by using the World Health Organization (WHO) approach and FP readiness indicators were identified according to the WHO Service Availability and Readiness Assessment (SARA) Manual. Through this approach, the FP service readiness indicators were categorized into three domains. The first domain included staff and guideline, which had two indicators, i.e., the guideline on FP and staff trained to provide FP services. The facilities with guidelines and at least one provider that had received in-service training in FP within 2 years were coded as “Yes,” while those without such guidelines or at least one provider had received in-service training in FP within 2 years were coded as “No.” The second domain was equipment, which had one indicator, i.e., the presence of blood pressure (BP) apparatus. Facilities with BP apparatus were coded as “Yes,” while those without such item were coded as “No.” The third domain was medicine and commodities, which had four indicators, i.e., the availabilities of progestin-only pills, combined oral pills, injectable contraceptives (combined or progestin-only), and condoms. Facilities with the availability of these methods of contraception were coded as “Yes,” while those without were coded as “No.” The FP readiness index was then summed by adding the presence of each indicator, with equal weight given to each of the domains and each of the indicators within the domains. As the target was 100%, each domain accounted for 33.3% (100%/3) of the index. The percentage for each indicator within the domain was equal to 33.3% divided by the number of indicators in that domain. The FP service readiness index for each facility was then calculated by adding the percentages. The similar approach has been used elsewhere [22]. The facilities with the score “less than 33.3%,” “between 33.3% and 66.7%,” and “more than 66.7%” were considered to have low, medium and high readiness for offering FP services respectively.

### Other independent variables (background factors)

These were categorized into provider characteristics (sex, qualification, and years of experience), facility characteristics (location, facility type, managing authority, routine management meetings and external supervision) and client characteristics (age and level of education).

### Statistical analysis

Data were analyzed using Stata 14 (StataCorp, College Texas). Since the facilities sampled were not evenly distributed and the response rate might be very different by regions or facility type, then over and under-sampled in the regions with fewer and more facilities respectively were performed before data collection. Therefore, before analysis, client weight was used to restore the actual representativeness and to correct for sampled data (weighting). Furthermore, the “SVY” set command was used to adjust for the complex sampling design employed in the TSPA survey so that to obtain the accurate estimates.

Descriptive statistics were presented as frequency listings and percentages because our variables are categorical. We fitted an unadjusted logistic regression model to assess whether there were any associations between the outcome and key independent variable, also, with other variables from the health facility, provider, and client separately. All variables with a *P*-value less than 0.2 were considered for inclusion into the multiple logistic regression (Adjusted) model using the stepwise (backward elimination) method to test for the association of each variable with the outcome variable. The obtained final model included all the variables that determined client satisfaction with FP services. The *P*-value < 0.05 and 95% confidence interval (CI) for the odds ratio (OR) were used to confirm the significance of the associations.

### Ethical considerations

The original 2014–2015 TSPA survey was approved by Tanzania’s National Institute for Medical Research (NIMR), the Zanzibar Medical Ethics and Research Committee (ZAMREC), and the Institutional Review Board of ICF International in the USA. The informed consent was obtained from the manager, the person-in-charge of the facility, or the most senior health worker responsible for client services present at the facility, and women received FP services within the facilities surveyed. The respondents were adequately informed regarding all relevant aspects of the study, including its aim and interview procedures.

## Results

### Background characteristics of observed consultations according to facilities, health providers, and clients

Out of the 1188 facilities included in the survey, 427 offered FP services with a total of 1746 observations and exit interviews. Majority of the FP consultations (1497, 85.7%) were performed in public facilities, more than half (954, 54.6%) were performed either in clinics or dispensaries, while 974 (55.8%) were performed in rural settings. About nine-tenths (1541, 88.3%) of the consultations were performed by female health providers. Furthermore, 1049 (63.5%) consolations were performed by providers with experience of 5 years or less, while only 55 (3.2%) consultations were performed by clinicians. The majority (931, 53.3%) of the consultations involved women between the age of 20 to 29 years (Table 1).

**Table 1:** Characteristics of health facilities, health providers and clients in the FP exit interviews (*n* = 1746)

Variable	Number (*n*)	Percentage (%)
Health facility characteristics		
Managing authority		
Public	1497	85.7
Private	249	14 3
Facility type		
Hospital	298	22.8
Health center	394	22.6
Clinic & dispensary	954	54.6
Facility location		
Urban	772	44.2
Rural	974	55.8
Routine management meetings		
No	214	12.2
Yes	1532	87.8
External supervision		
Not done	359	20.6
Yes, < 3 months	1052	60.2
Yes, > 3 months	335	19.2
Health provider characteristics		
Sex of provider		
Male	205	11.7
Female	1541	88.3
Working experience (years)^a^		
0–5	1048	63.5
> 5	696	36.5
Qualification		
Clinicians	55	3.2
Nurses	1691	96.8
Client characteristics		
Age		
< 20	158	9.1
20–29	931	53.3
30–39	535	30.7
> =40	121	6.9
Formal education		
No	280	16.1
Yes	1466	83.9
Level of formal education^b^		
Primary	1221	83.4
Secondary	223	15.2
Tertiary	22	1.4

^a^Do not add up to 1746 because some providers did not remember the year they started working in the facility

^b^Do not add up to 1746 because some clients did not have any formal education

### Client satisfaction with FP services

Out of 1746 observed FP consultations, the majority (1592, 91%) of consultations clients reported that had satisfied with the services received or provided to them; only 153 (9%) of the consultations the clients reported not being satisfied with the services**.**

### Indicators of service readiness by managing authority

**Fig. 1:**
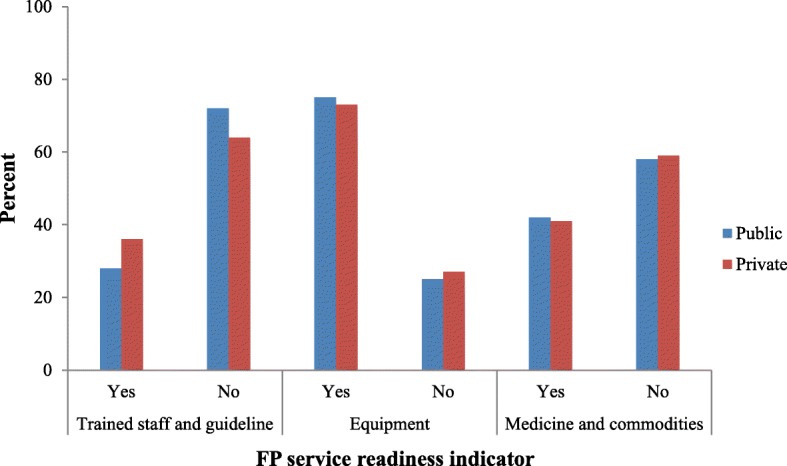
Individual indicators of service readiness by managing authority

About third-quarters of public (75.1%) and private (73.3%) facilities reported having equipment for providing FP services while only 42% stocked contraceptives (e.g. oral pills, injectable contraceptives and/or condoms). Further, trained staff and clinical guidelines were present in only 30% of services. When the indicators for service readiness were compared by managing authority, it was found that availability of the indicators was comparable between public and privately owned facilities. A major difference was in the presence of trained staff and clinical guideline that was high in private (36%) than public (28%) facilities (*P* < 0.05). However, when the overall FP service readiness was compared according to managing authority, the scores were comparable between public and privately owned facilities, especially in the medium and high scores categories. In the low category of service readiness, public facilities were found to perform poor (7.1%) than private facilities (11.2%) (Fig. 1).

### Factors associated with client satisfaction

Table 2 presents the results of unadjusted and adjusted analyses. In adjusted analysis, high FP service readiness and receiving services from private owned facilities were significantly associated with client satisfaction with FP services [AOR = 2.5; 95% CI, 1.1–6.0] and [AOR = 2.3; 95% CI, 1.1–5.0] respectively. Additionally, compared with clients who were < 20 years, clients with the age between 20 to 29 years were significantly less likely to be satisfied with FP services [AOR = 0.3; 95% CI, 0.1–0.7]**.**

**Table 2:** Bivariate and Multivariate analysis of factors associated with client satisfaction to family planning services

Variable	Client satisfied Number (%)	Unadjusted OR [95% CI]	Adjusted OR [95% CI]
Service readiness			
Low	163 (10.2)	Ref.	Ref.
Medium	1079 (67.8)	1.3 [0.6–2.7]	1.3 [0.6–2.7]
High	351 (22.0)	2.7 [1.1–6.3]	2.5 [1.1–6.0]
Managing authority			
Public	1355 (85.1)	Ref.	Ref.
Private	238 (14.9)	2.1 [0.9–4.9]	2.3 [1.1–5.0]
Facility type			
Hospital	362 (22.7)	Ref.	Ref.
Health center	348 (21.9)	0.8 [0.5–1.2]	0.8 [0.4–1.5]
Clinic & dispensary	883 (55.4)	1.2 [0.7–2.3]	1.2 [0.5–2.3]
Facility location			
Rural	887 (55.7)	Ref.	Ref.
Urban	706 (44.3)	1.1 [0.6–1.7]	1.1 [0.6–2.0]
Routine management meetings			
No	194 (12.2)	Ref.	Ref.
Yes	1399 (87.8)	1.1 [0.4–3.1]	1.1 [0.4–3.4]
External supervision			
No	323 (20.3)	Ref.	Ref.
Yes, < 3 months	955 (59.9)	1.1 [0.6–2.0]	1.2 [0.7–2.2]
Yes, > 3 months	315 (19.8)	1.8 [0.9–3.8].	2.0 [0.9–4.2]
Sex of the Provider			
Male	1403 (88.1)	Ref.	Ref.
Female	190 (11.9)	1.3 [0.6–2.6]	1.2 [0.5–2.8]
Working experience			
5 years and less	961 (60.4)	Ref.	Ref.
More than 5 years	630 (39.6)	0.9 [0.5–1.4]	0.9 [0.5–1.3]
Qualification			
Clinician	51 (3.2)	Ref.	Ref.
Nurses	1542 (96.8)	0.8 [0.3–2.6]	0.8 [0.3–2.8]
Age of client			
< 20	153 (9.6)	Ref.	Ref.
20–29	834 (52.4)	0.3 [0.1–0.7]	0.3 [0.1–0.7]
30–39	495 (31.0)	0.4 [0.2–0.9]	0.4 [0.2–1.1]
> =40	111 (7.0)	0.4 [0.1–1.4]	0.4 [0.1–1.5]
Education			
No	258 (16.2)	Ref.	Ref.
Yes	1335 (83.2)	0.9 [0.5–1.6]	0.9 [0.5–1.7]

## Discussion

This study aimed to examine the current status of and factors associated with client satisfaction with FP services in Tanzania, which has a high unmet need for FP. It found the high proportion of women had satisfied with the FP services. In addition, FP service readiness and its individual indicators except for trained staff and clinical guideline were comparable between the publicly and privately-owned facilities. Furthermore, high FP service readiness and privately-owned facilities were significantly associated with client’s satisfaction with FP services.

The high proportion of client’s satisfaction with FP services observed in this study was higher than earlier studies performed in Tanzania [23], South Ethiopia [3], Nigeria [19], and Kenya but comparable to the study performed in Mozambique [20]. The high proportion of clients’ satisfaction with FP services in the current study can be explained by the increased number of health facilities that provide FP services compared to before, provision of in-service training to health care providers and important supplies (contraceptives and commodities) from governmental and some non-governmental organizations that operate in Tanzania. Also, the study found no significant difference in FP service readiness between private and public facilities. This finding was in agreement to that of the previous study performed in Tanzania using the 2006 Service Provision Assessment survey [23].

Understanding the factors associated with client’s satisfaction with services received or provided could help the health providers and policy-decision makers to design programmes that will meet the client needs [24]. This study revealed that client’s satisfaction with services was associated with a high FP service readiness scores. Due to lack of studies that assessed the association between FP service readiness and clients’ satisfaction with FP services it becomes difficult to compare this finding. However, previous studies performed in Kenya and Mozambique found that availability of essential equipment as the important domain of FP service readiness was associated with client’s satisfaction [18, 20]. These findings are not in agreement with the findings from the previous study that did not find any association between FP service readiness and client’s satisfaction [10]. The difference in findings might be due to that the previous survey analyzed data that were collected more than 10 years ago, while the current study analyzed data that collected 2014–2015. Therefore ten-year deference in this era of globalization could explain the change of some of the socio-economic and demographic factors.

Evidence from the previous study suggests that client’s satisfaction differs by type of managing authority [25]. In agreement with this, the current study found that the odds of clients being satisfied with FP services were two times higher for privately-owned facilities than publicly-owned one. These findings are in agreement with those of previous studies performed in Kenya [10, 18, 25, 26] and in West African countries [27]. The similarity of these findings might be explained by the fact that privately-owned facilities are more responsible and committed to the patients since are operated for profit basis compared to publicly-owned facilities, therefore, tend to provide better and complete services in order to gain more clients and profit [28]. Additionally, privately-owned facilities are not crowded and clients could quickly receive service and spent plenty of time with health providers during consultations hence more likely to be satisfied. On the other hand, publicly-owned facilities are characterized with the long queue, staffs that have low morale or relatively poor performance and attendance [29]. These facilities usually have the shortage of staff, medicines and functioning equipment required to provide standard services [30, 31].

The current study found that clients in the age between 20 to 29 years were less likely to report being satisfied with FP services compared to those less than 20 years. This observation was in agreement with those of the previous studies performed in Kenya and Egypt [18, 32]. This finding might be explained by the fact that younger clients are more likely to have more expectations compared to older ones; also, younger clients are more likely to be new FP clients with first-time visits that do not know components of FP services that should be provided to them. One previous study found that repeat FP clients were more likely to be satisfied than first-time clients [3]. Other studies, however, have found no association between clients’ age and satisfaction with FP services [3, 27, 33, 34].

Unlike in other previous studies performed elsewhere, that found an association between clients’ level of education and satisfaction with FP services, the current study found no association between those variables. Some of those previous studies found that clients with the higher level of education were more likely to be satisfied with the services they receive [10, 33], while others found that less educated ones were more likely to be satisfied [18]. Furthermore, the current study found that providers’ years of experience were not significantly associated with client satisfaction. This finding was in contrast to those of previous studies performed in Kenya and Senegal [18, 34]. However, the current study found that facility type and location were not associated with client’s satisfaction. Similar findings have been reported in previous studies conducted in Ethiopia, Nigeria, and Mozambique [3, 19, 20].

The strength of the current study is that it analyzed the data with a nationwide representative sample of health facilities, with a response rate of 99%. The use of such data suggests that the findings accurately reflect the current situation regarding FP services in the study area. By considering the complex sampling techniques involved in the TSPA survey, the findings were adjusted for clustering effect and weighted to correct for non- response and disproportionate sampling. However, the study has some limitations, due to its cross-sectional nature, it fails to explain the causality assumptions hence results should be interpreted with caution. Additionally, the outcome variable “client satisfaction” in the current study was assessed by using a single question with response of “very satisfied,” “more or less satisfied,” and “not satisfied”, this might be not very sensitive enough that is why we found high proportion of client who was satisfied with FP services. Furthermore, since exit interviews were based on prior direct observations between client and provider, our findings might be subjected to social desirability bias, observation bias, and Hawthorne effect; that is the change of behavior by the subjects of a study due to their awareness of being observed [35]. However, we minimized these biases by restricted to a maximum of five observations per each provider.

## Conclusion

There is a high level of client satisfaction with FP services in Tanzania. High FP service readiness score, privately-owned facilities, and client’s age matter on client’s satisfaction with FP services. Maintaining and exceeding this level will require improvements in the provision of staff training and the availability of contraceptives in existing services.

## Abbreviations

AOR: Adjusted odds ratio
BP: Blood pressure
COR: Crude odds ratio
DHS’: Demographic health survey
FP: Family planning
MOHCDGEC: Ministry of Health, Community Development, Gender, Elderly and Children
NFPCIP: National family planning costed implementation program
SARA: Service availability and readiness assessment
SSA: Sub-Saharan Africa
TSPA: Tanzania service provision assessment
WHO: World Health Organization
